# Neuromuscular Electrical Stimulation Induces Skeletal Muscle Fiber Remodeling and Specific Gene Expression Profile in Healthy Elderly

**DOI:** 10.3389/fphys.2019.01459

**Published:** 2019-11-27

**Authors:** Rosa Mancinelli, Luana Toniolo, Ester Sara Di Filippo, Christian Doria, Mariangela Marrone, Camilla Reina Maroni, Vittore Verratti, Danilo Bondi, Lisa Maccatrozzo, Tiziana Pietrangelo, Stefania Fulle

**Affiliations:** ^1^Department of Neuroscience Imaging and Clinical Sciences, ‘G. d’Annunzio’ University of Chieti–Pescara, Chieti, Italy; ^2^Interuniversity Institute of Myology, Rome, Italy; ^3^Laboratory of Functional Evaluation, ‘G. d’Annunzio’ University of Chieti–Pescara, Chieti, Italy; ^4^Department of Biomedical Sciences, University of Padova, Padua, Italy; ^5^Department of Psychological, Health and Territorial Sciences, ‘G. d’Annunzio’ University of Chieti–Pescara, Chieti, Italy; ^6^Department of Comparative Biomedicine and Food Science, University of Padova, Padua, Italy

**Keywords:** oxidative stress, maximal voluntary contraction, neuromuscular electrical stimulation, single fiber mechanic, gene expression

## Abstract

Skeletal muscle aging is a multifactorial process strictly related to progressive weakness. One of the results that were focused on was the fiber phenotype modification and their loss. The physiological muscle recruitment to contraction, basically prosecuted under volitional control, can also be engaged by means of Neuromuscular Electrical Stimulation (NMES). Knowing that the NMES is effective in improving muscle strength in active healthy elderly, the aim was to investigate which physiological modifications were able to produce in the *Vastus lateralis* muscle and the pathways involved. It was found that NMES increased the cross sectional area and the isometric strength of type II myofibers together with the activated myogenic pathway in order to shift glycolytic toward the oxidative phenotype II myofibers, at a molecular level and with an increase of maximal voluntary contraction (MVC) at a functional level. Using the TaqMan low density array on 48 different genes, we found that NMES specific gene regulation highlighted: (i) increased protein synthesis with respect to protein degradation; (ii) the activation of an apoptotic pathway involved in the differentiation process; (iii) increased regeneration signals; (iv) oxidative enzyme regulation. These pathways were validated via confirmatory RT-PCR for genes involved in the regeneration process as well as Myosin isoforms. We also investigated the oxidative stress status analyzing superoxide anion levels, the protein expression of two different superoxide dismutase and the activity of both catalase and superoxide anion dismutase, being two main antioxidant enzymes. In conclusion, data demonstrates that NMES is effective in producing physiological adaptation on *Vastus Lateralis* of active healthy elderly as well as providing new insights for further research on elderly who experienced muscle detriment for periodic or permanent immobility.

## Introduction

Skeletal muscle is a tissue of our organism that undergoes adaptations. However, during aging it is related to the progressive loss of the neuromuscular function that takes the name of Sarcopenia. This term describes a condition characterized by the loss of skeletal muscle strength and mass that occurs during aging (Barber et al., 2015). Sarcopenia increases in those above 60 years of age with atrophy being an important symptom (Pietrangelo et al., 2009) linked to, a reduction of hormonal levels (testosterone, GH, IGF-1), sedentary lifestyle, genetic and reduced regenerative capability-stem cells dependent on skeletal muscle (Cruz-Jentoft et al., 2019).

Skeletal muscle atrophy in the elderly is worsened by inactivity that occurs when diseases obligate them to be bed-ridden. The best countermeasure is moderate and regular exercise, despite the fact that it could be useful, there is a lack of precise indications with regards to specific training and/or the treatment of Sarcopenia (Stec et al., 2017; Steffl et al., 2017).

To date, muscle strengthening against resistance is the most widely used training protocol applied in order to avoid loss of muscle strength and mass, that occurs with aging. It has been demonstrated that in the elderly, this kind of training protocol leads to increased protein synthesis associated with muscle strength (Kosek et al., 2006). Nevertheless, one of the main problems related to aging is that some people are not able to move because of pathological conditions such as pain, osteoarthritis, scarce motivation and limited motor skills that reduce the execution of classic training protocols. Neuromuscular Electrical Stimulation (NMES) can be considered as an alternative approach in place of physical exercise mimicking the same effect. Indeed, NMES is a fine tool to counteract the onset or aggravation of the sarcopenia process activating the plasticity of muscular tissue (Dehail et al., 2008). The NMES can be utilized to counteract the progression of muscle weakness due to injury or knee surgery in medicine (Rebai et al., 2002; Stevens et al., 2003; Talbot et al., 2003) as well as increase muscle strength and hypertrophy in healthy subjects (Yanagi et al., 2003; Minetto et al., 2013; Di Filippo et al., 2017). Few studies have analyzed the effects of NMES on the functionality of muscle in elderly subjects *in vivo*; furthermore, few data are available regarding changes induced by NMES in single myofibers dissociated from aged muscle (Di Filippo et al., 2017). Studying the effects and the mechanisms activated by NMES will give indications in how to use this type of training in sarcopenic subjects, especially in the elderly who are not capable of doing voluntary exercise. The analysis of NMES training could offer significant advantages in understanding if these protocols *per se* or in association with voluntary training could defer sarcopenia in elderly people (D’Antona et al., 2003; Maffiuletti et al., 2006). Moreover, very little evidence exists with regards to oxidative management in elderly muscles stimulated with NMES. Few studies have addressed this topic on young males. Gondin et al. (2011) showed that in young males NMES improved the antioxidant defense system. Some evidence at cellular level, suggests that electrical stimulation increases the ROS production (Dong et al., 2018). Our group recently stated that NMES can influence the regeneration process as well as the oxidative stress of satellite cells in human skeletal muscle (Di Filippo et al., 2017). However, oxidative management in NMES-stimulated muscle tissue of elderly still remains to be further investigated.

The goal of the present study was to determine the adaptation of skeletal muscle tissue/myofibers especially at a molecular level as well as oxidative modulation, by using a passive muscle training program such as NMES which is applied to the quadriceps muscles related to increase muscle strength and mass in elderly subjects without any voluntary muscle contraction.

The impact of NMES on local muscle in elderly volunteers was assessed both by isometric strength developed in MVCs by thigh extensor muscles and thigh circumference. Structural modifications were evaluated using thigh circumference parameters. In particular, *Vastus Lateralis* (VL) muscle needle-biopsies obtained pre and post-NMES were used to analyze specific fiber features (cross-sectional area, types, tension development of single fiber) and the expression of specific groups of genes.

## Materials and Methods

### Subjects

The study was carried out on 18 healthy elderly male subjects (67.63 ± 4.94 years old, mean ± SD). The study was approved by the local Ethics Committee (protocol nos. 1233/06, 1884/09 and 07/2016 COET), and was in accordance to the 1964 Declaration of Helsinki. All subjects provided written informed consent before participating in the research project. The following inclusion criteria have been taken into account: normal blood pressure and ECG; the absence of cardiovascular, metabolic and bone/joint diseases. Exclusion criteria were considered the presence of cardiovascular and/or metabolic diseases, evidence of acquired or hereditary muscle disease, diagnosis of respiratory or psychiatric disorders. No individual was under treatment with testosterone or other pharmacological therapies nor training protocols known to influence skeletal muscle.

### NMES Protocol and Experimental Design

Neuromuscular Electrical Stimulation sessions consisted of 24 sessions of bilateral stimulation lasting 18-min each with three sessions per week according to the modified methods of Maffiuletti (2010) and Di Filippo et al. (2017). During stimulation, subjects were seated with the knee joint fixed at a 75° angle (where 0° corresponds to a full knee extension). To minimize hip and thigh motion during contractions, straps were firmly fastened across the pelvis. Three self-adhesive electrodes of 2-mmwide were placed over each thigh. Two positive electrodes (25 cm^2^) were placed as close as possible to the motor point of both the *Vastus Lateralis* and *Vastus Medialis* muscles. The negative electrode (50 cm^2^), was placed 5–7 cm below the inguinal crease. The NMES device was a portable battery-powered stimulator (Genesy 1200 Pro, Globus^®^ Srl, Codognè, TV, Italy). Rectangular wave pulsed currents (75 Hz) lasting 400 μs were delivered with a rise time of 1.5 s, a steady tetanic stimulation time of 4 s, and a fall time of 0.75 s, for a total contraction duration of 6.25 s followed by a pause, lasting 20 s. The duty cycle was 24% (6.25/26.25 s of work divided by seconds of the total work). Intensity was monitored online and was gradually increased throughout the training session to a level of maximized tolerance intensity. Each session was preceded by a standardized warmup consisting of 5 Hz pulses lasting 200 μs. Furthermore, the intensity of the stimulation was monitored and recorded up to the individual’s pain threshold.

### Anthropometric Data

The pre-NMES session (1 week before the stimulation) and the post-NMES session (one-three days after the completion of the stimulation), the subjects were characterized for Body Mass Index (BMI) and the circumference of the dominant lower limbs measured at superior, intermediate and inferior levels as well as at the linea glutea (Pietrangelo et al., 2011, 2012).

### Maximal Isometric Strength

The maximal isometric strength of the lower limbs was determined by measuring the MVC according to Pietrangelo et al. (2012). The tests were carried out (with) a leg-extension device (Nessfit Srl, San Giovanni Teatino, Italy) equipped with a strain gauge (Globus, Codognè, Italy), repeated three times, with a 2 min recovery time between each). The knees and body/limb joints were positioned at 90°. The valid MVC was the highest value recorded.

### Molecular Analysis of Muscle Biopsies

Using a semi-automatic needle (Precisa 13 Gauge; Hospital Service, Rome, Italy) at pre- and post-NMES, Vastus lateralis muscle biopsies were obtained at one-third of the distance from the upper margin of the rotula to the anterior superior iliac spine as described in Pietrangelo (Pietrangelo et al., 2011). In each subject, several samples were collected from the same insertion point of the needle and were divided into three groups: (i) samples of approximately 10 mg collected in RNA Later (#AM7020, Ambion, Milan, Italy), and stored at −80°C until used to perform the RT-PCR Analysis, (ii) samples collected in ice cold skinning solution with 50% (v/v) glycerol and stored at –20°C for myofibers preservation and Electrophoretic Analysis, (iii) samples immediately frozen and stored at −80°C for enzymatic and Western Blotting Analysis.

### Real-Time PCR

Total purified RNA (by TRIZOL Reagent from Invitrogen, Thermo Fisher Scientific) was quantified using NanoDrop Spectrophotometers (Thermo Fisher Scientific) and RNA quality was evaluated by gel electrophoresis according to Boscolo Papo et al. (2014). The cDNA was synthesized from 1 μg of the total RNA using the High Capacity cDNA Reverse Transcription Kit with RNase Inhibitor (Applied Biosystems, Thermo Fisher Scientific), in accordance to the manufacturer’s protocol. Each sample was used to perform both the classic RT-PCR and TaqMan low density array.

The expression analysis using the classic RT-PCR was performed using the ABI 7500 Real-Time PCR System (Applied Biosystems, Thermo Fisher Scientific). The data were acquired by ABI’s 7500 System SDS Software. The SYBR Green I dye chemistry detection was used to amplify myogenic regulatory factors (IGF1, MURF1, Pax7, and MSTN) and the TaqMan Assay were used to amplify myosin isoforms (MyHC 1, MyHC 2A, and MyHC 2X). Quantitative real time PCR was performed in 20 μl reaction volume containing 1X Power SybrGreen or TaqMan Gene Expression PCR Master Mix (Applied Biosystem, Thermo Fisher Scientific), 300 nM each forward and reverse primers and 100 ng of cDNA. Dissociation curves confirmed the specific amplification of the cDNA target and the absence of non-specific products.

The expression analysis using TaqMan low density array (Applied Biosystems-MDS Sciex, Toronto, ON, Canada) was performed on 100 ng (2 μl) cDNA for each sample according to Fulle et al. (2013). Subsequently, 48 μl nuclease-free water and 50 μl 2× TaqMan Universal PCR Master Mix (Applied Biosystems) were added for the RT-PCR measurements. This mixture was divided over sample-loading ports of the TaqMan low density arrays. The arrays were centrifuged twice (2 min, 331 × *g* at room temperature) and then, the card sealed. The amplification of Real-time PCR was performed using an Applied Biosystems Prism 7900HT Sequence Detection System, connected to the Sequence Detector Software (SDS version 2.0; Applied Biosystems) for data collection and subsequent analysis.

For this purpose, we chose arrays preloaded with 48 selected genes related to the following pathways: myogenesis, apoptosis, protein anabolism/catabolism and antioxidant enzymes; each array was useful to assess eight different samples.

For both classic RT-PCR and TaqMan low density array, the relative quantification of target gene expression was evaluated with data derived from the SDS software, utilizing the arithmetical formula 2^–ΔΔCt^, according to the comparative Ct method as reported in Di Filippo et al. (2016). The data have been deposited in NCBI’s Gene Expression Omnibus and are accessible through GEO Series accession number GSE133720^1^.

### Mechanical Characterization of Vastus Lateralis Myofibers

The mechanical characterization of single myofibers was performed according to Di Filippo et al. (2017). Briefly, muscle biopsy fragments were stored in skinning solution until analyzed. Then, the solution was replaced with ATP, single fibers were dissected, bathed in an appropriate solution and transferred to the experimental apparatus where all parameters were measured. We tested 216 fibers.

After the mechanical measurements, the myofibers were classified according to their MyHC isoform composition which was determined by gel electrophoresis (Venturelli et al., 2015). Shortly after, appropriate amounts of protein were diluted in an appropriate solution, boiled and loaded onto a gel. Separation was performed and the following staining identified the bands corresponding to the MHC isoforms.

### NBT Assay

The NBT (Nitro Blue Tetrazolium chloride, SIGMA-Aldrich, Milan, Italy) assay is conventionally used to evaluate the production of $O_{2}^{•-}$. It is a spectrophotometric assay, based on the reduction of NBT in Nitro blue-formazan in the presence of $O_{2}^{•-}$, and was performed on skeletal muscle tissue biopsies.

### Protein Isolation and Quantization

Antioxidant enzyme assays and Western Blotting were performed, according to Marrone (Marrone et al., 2018) using muscles homogenized in 100 mM Na-phosphate buffer pH 7.0 with 1:100 protease inhibitors cocktail (#P8340, Sigma-Aldrich) and centrifuged at 10,000 × *g* for 15 min at 4°C. Protein concentrations were measured on the deriving supernatant according to the Bradford method (Bradford Reagent, #B6916, Sigma-Aldrich).

### Superoxide Enzyme Activities

The activity of Superoxide Dismutase 1 (SOD1) was determined by using the modified method of L’Abbe’ and Fischer (Marrone et al., 2018). The final assay volume of 1 ml contained 20 mM Na_2_CO_3_, pH 10, 10 mM cytochrome c, 1 mM xantine and xantine oxidase. As the xanthine oxidase activity varies, the amount used for the assay was sufficient to stimulate a cytochrome c reduction at 550 nm at a rate of 0.025 per minute without SOD addition. SOD units were calculated on the basis of the definition that one unit represents the activity that inhibits the cytochrome c reduction by 50%.

### Catalase Activity

Catalase activity was determined, as previously described in Shakirzyanova (Shakirzyanova et al., 2016) by evaluating the decrease in absorbance due to H_2_O_2_ consumption (ϵ = 0.04 mM^–1^ cm^–1^) measured at a wavelength of 240 nm. 1 ml of the final reaction mixture contained 100 mM Na-phosphate buffer pH 7.0, 12 mM H_2_O_2_ and 70 μg sample.

### Western Blotting Analysis

Western Blotting Analysis was performed according to Marrone (Marrone et al., 2018) on 30 μg lysates from fragments of old *Vastus Lateralis* skeletal muscle pre- and post-NMES, using SOD1 (71G8) mouse mAb (#4266, Cell Signaling Technology, Danvers, MA, United States) at 1:1000, SOD2 (D9V9C) rabbit mAb (#13194, Cell signaling Technology) at 1:1000, GAPDH (6C5) sc-32233 mouse (Santa Cruz Biotechnology, INC) at 1:600, as a primary antibody. Secondary HRP-conjugated antibodies (Cell Signaling Technology) at 1:5000. Bands were detected and pictured at Uvitec (Cambridge, United Kingdom) by Amersham ECL Prime Western Blotting Detection Reagent (#RPN2236, GE Healthcare); densitometry analyses were performed with ImageJ software (Marrone et al., 2018).

### Statistical Analysis

The statistical analysis of muscle myofibers was carried out using GraphPad Prism Software, version 7 (GraphPad Software, La Jolla, United States) and R-based open source software Jamovi^2^. The normality of distribution was assessed by D’Agostino–Pearson Omnibus Test and Shapiro–Wilk Test.

The Repeated Measures ANOVA (between factor: NMES *vs.* Control) was conducted to analyze the anthropometric and strength parameters.

The CSA values then underwent logarithmic transformation, while F_0_ and P_0_ underwent a square root transformation. Identification of outliers was performed with the ROUT Method (*Q* = 0.5%). General Linear Mixed Model (GLMM) with Restricted Maximum Likelihood (REML) estimation method was used to analyze differences, setting pre-post and fiber typology as fixed effects and subjects as random effect.

The statistical analysis of NBT, enzymatic activity assays and densitometric analysis of Western Blotting were performed with GraphPad Prism Software and an unpaired *t*-test.

## Results

### Effects of NMES on Anthropometric Data

The anthropometric characteristics of healthy male subjects did not change when comparing pre- and post-NMES conditions (Table 1).

**TABLE 1:** Anthropometric and functional characteristics of healthy elderly subjects.

**Characteristics**	**pre-NMES**	**post-NMES**	**Pre-control**	**Post-control**
Weight (kg)	75.1 ± 8.1	75.2 ± 7.9	76.0 ± 6.0	76.5 ± 5.8
BMI (kg m^–2^)	27.7 ± 3.0	27.7 ± 3.1	28.8 ± 3.0	28.9 ± 2.9
Body fat (%)	26.6 ± 5.3	25.4 ± 4.9	27.0 ± 4.8	26.8 ± 4.3
Circ. Sup. (cm)	53.8 ± 3.0	54.1 ± 3.1	53.2 ± 2.5	53.5 ± 2.5
Circ. Inter. (cm)	47.0 ± 2.7	47.7 ± 2.8	45.8 ± 2.1	46.5 ± 1.8
Circ. Inf. (cm)	40.5 ± 2.9	41.2 ± 3.1	38.9 ± 1.9	39.1 ± 2.3
MVC_bil_ (N)	537 ± 104	585 ± 111^∗^	488 ± 133	492 ± 116

*Control subject (*n* = 10; 70.8 ± 3.08 years old); BMI, body mass index; Cir, circumference; sup, superior; inter, inter-medium; inf, inferior; MVC_*bil*_, maximal bilateral voluntary contraction, ^∗^*p* < 0.05. The data are expressed as mean ± standard deviation.*

### Effects of NMES on the Strength of the Elderly

The isometric strength measured on lower limbs revealed that the post-NMES MVC was significantly increased (Table 1) with respect to pre-NMES (bilateral, *p* < 0.05).

### Muscle Fiber Cross-Sectional Area, Strength and Specific Tension on Different Fiber Phenotypes

The results on all fiber type analysis showed that the CSA increased at post-NMES in respect to pre-NMES (3,720 ± 222 μm^2^ *vs.* 3,700 ± 184 μm^2^, *p* = 0.075). Specifically, the myofibers IIa significantly increased their CSA (*p* < 0.05, Table 2). The interaction fiber x NMES ex-post showed a strong tendency, reflecting a different trend of different myofibers (*p* = 0.053).

**TABLE 2:** Statistical parameters of general linear mixed model relative to skeletal muscle myofiber results showed in Figure 1.

**Samples**	**CSA**	***F*_0_**	***P*_0_**
NMES pre-post	*p* = 0.075	*p* < 0.001	*p* = 0.072
Myofibers	*p* = 0.087	*p* = 0.256	*p* = 0.001
Protocol x myofibers	*p* = 0.053	*p* = 0.338	*p* = 0.939
*R*^2^	0.07	0.04	0.100
*R*^2^ conditional	0.19	0.64	0.110
**Random effect**			
Intraclass coefficient	0.134	0.629	0.011
Likelihood ratio test	*p* = 0.001	*p* < 0.001	*p* = 0.560
**Simple effect PRE-POST**			
Myofibers I	*p* = 0.219	*p* = 0.158	*p* = 0.058
Myofibers IIa	*p* = 0.046	*p* = 0.012	*p* = 0.290
Myofibers IIax	*p* = 0.670	*p* = 0.019	*p* = 0.234
Myofibers IIx	*p* = 0.088	*p* = 0.044	*p* = 0.769

*CSA, cross sectional area; F_0_, isometric force of single fiber; P_0_, specific tension as force divided per CSA of single fibers.*

**FIGURE 1:**
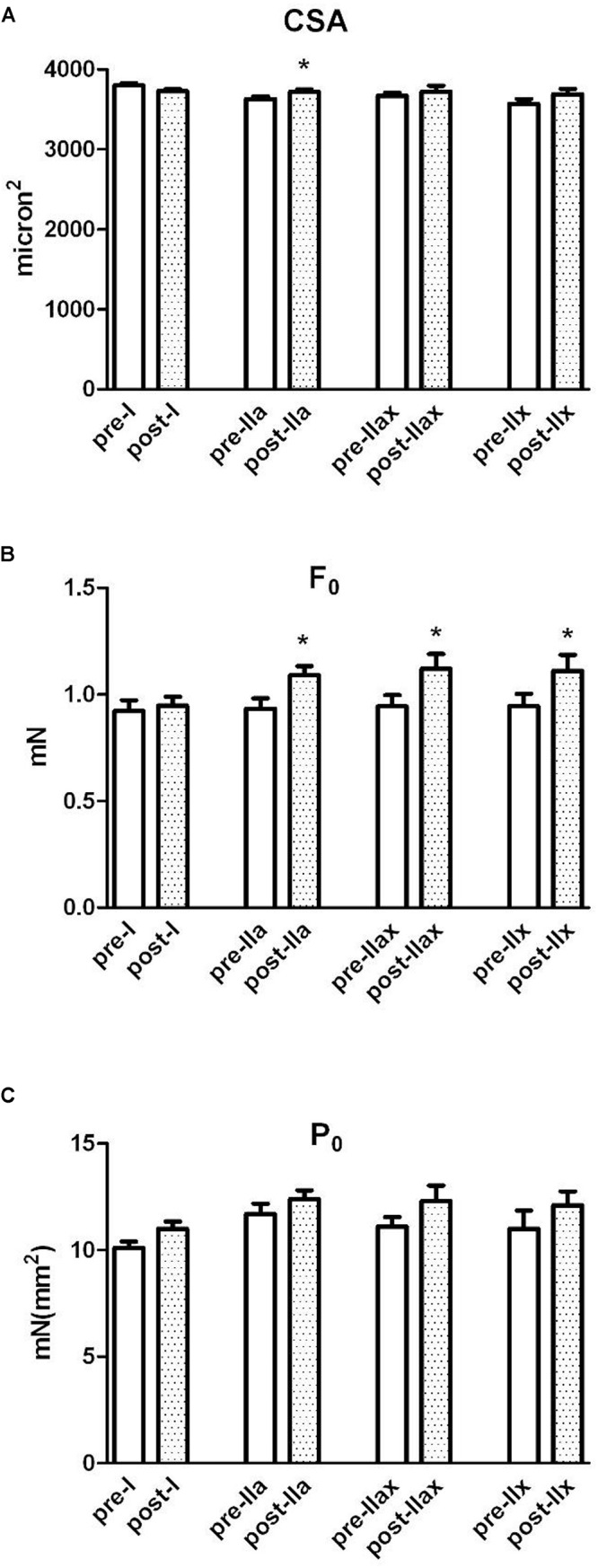
Single-fiber analysis, cross sectional area, force and specific isometric tension. The white and dotted columns represent, respectively, pre-NMES and post-NMES samples divided in fiber types (I, IIa, IIax, IIx). **(A)** Shows the cross-sectional area (CSA) of single muscle myofibers obtained by the *Vastus Lateralis*. **(B)** Shows the force developed by single fibers. **(C)** Reports measurements of specific isometric tension (force/CSA) developed in maximal calcium activated contraction by the same myofibers. ^∗^*p* < 0.05.

The results on single muscle myofibers divided into typologies are displayed in Figure 1.

The average force (F_0_) significantly increased approximately 10%, from 0.932 ± 0.281 mN to 1.02 ± 0.281 in post-NMES (*p* < 0.001). Specifically, the myofibers II (type a, ax, and x) significantly incased their F_0_ as shown in Figure 1 (*p* < 0.05 Table 2).

The value of specific tension (P_0_), the isometric strength per unit of fiber area (F_o_/CSA), was 10.9 ± 2.38 mN mm^–2^ and 11.6 ± 2.38 mN mm^–2^ in pre- and post-NMES, respectively. Though P_*o*_ tended to increase and this increment was not statistically significant, it is worth mentioning that among the myofiber types, myofibers I showed this tendency (*p* = 0.058, Table 2).

As can be seen in Table 1, weight, BMI and % of body fat did not vary significantly, while the strength increased significantly at the end of the session (^∗^*p* < 0.05).

### Muscle Fiber CSA and Specific Tension

Figure 1 showed three parameters measured by single fiber mechanic experiments. The myofibers were grouped in type I, IIa, IIax, IIx. The empty bars indicate the pre-NMES samples, while the dotted bars indicate the post-NMES samples of the same volunteers.

The CSA (panel A), resulted to have increased at post-NMES specifically on myofibers IIa (*p* < 0.05, and also reported in the second line of Simple effect PRE-POST paragraph in Table 2). Panel B reported the F_0_ values, that significantly increased in myofibers IIa, IIax, IIx (*p* < 0.05, and also reported in the second, third and fourth line of Simple effect PRE-POST paragraph in Table 2). Panel C, reported the P_0_ value, that showed a tendency to increase only on fiber I (*p* = 0.058 reported in second, third and fourth line of Simple effect PRE-POST paragraph in Table 2).

Table 2 reported the statistical GLMM parameters used for mechanical experiments that allowed us to test data as a random effect.

### Gene Expression, TaqMan Low Density Array

Table 3 shows genes found significantly up and downregulated in post-NMES *versus* pre-NMES samples among 48 genes tested. Genes and their expression levels expressed as the log_10_ of Relative Quantifications (RQ) related to protein balance, oxidative management, apoptosis and myogenesis pathways, were analyzed with Real Time PCR using TaqMan low density arrays.

**TABLE 3:** Significantly dysregulated genes on skeletal muscle after NMES.

**Gene name**	**Gene symbol**	**Mean log_10_ RQ ± SE**
**Protein balance**		
Insulin like growth factor 1	IGF1	0.36 ± 0.18
Phosphatidylinositol-4,5-bisphosphate 3-kinase catalytic subunit alpha	PI3KCA	0.12 ± 0.06
Mechanistic target of rapamycin	MTOR	0.17 ± 0.11
Forkhead box O1	FOXO1	−0.28 ± 0.15
Myostatin	MSTN	−0.15 ± 0.13
AKT serine/threonine kinase	AKT	0.05 ± 0.11
Mitochondrial E3 ubiquitin protein ligase 1	MUL1	−0.03 ± 0.05
Ubiquitin like modifier activating enzyme 1	UBA1	0.01 ± 0.11
Ubiquitin conjugating enzyme E2 A	UBE2A	−0.02 ± 0.17
Tripartite motif containing 63	TRIM63 (MURF1)	−0.06 ± 0.12
Proteasome 26S subunit, ATPase 6	PSMC6	−0.20 ± 0.23
**Oxidative management**		
Catalase	CAT	−0.03 ± 0.09
Superoxide dismutase 1, soluble	SOD1	0.00 ± 0.14
Superoxide dismutase 2, mitochondrial	SOD2	−0.12 ± 0.09
Glutathione peroxidase 1	GPX1	−0.39 ± 0.17
Glutathione-disulfide reductase	GSR	0.12 ± 0.10
Glutathione S-transferase kappa 1	GSTK1	−0.18 ± 0.19
**Apoptosis**		
BCL2 associated agonist of cell death	BAD	−0.42 ± 0.33
BCL2, apoptosis regulator	BCL2	−0.31 ± 0.09
Caspase 2	CASP2	0.43 ± 0.09
Caspase 3	CASP3	−0.03 ± 0.29
Caspase 6	CASP6	0.08 ± 0.15
Caspase 7	CASP7	0.24 ± 0.09
Caspase 8	CASP8	0.30 ± 0.30
Caspase 9	CASP9	−0.17 ± 0.71
Tumor necrosis factor	TNF	0.32 ± 0.32
Fas associated via death domain	FADD	−0.03 ± 0.31
**Myogenesis**		
Myogenic differentiation 1	MYOD1	0.24 ± 0.11
Paired box 7	PAX7	0.06 ± 0.14
Tumor necrosis factor	TNF	0.32 ± 0.32
Mitogen-activated protein kinase 1	MAPK1 (p38)	0.17 ± 0.07
Myogenin	MYOG	0.16 ± 0.11

*Gene expression levels were analyzed with RT-PCR using TaqMan low density arrays. Data are expressed as the log_10_ of Relative Quantification (RQ) of the transcripts for the target genes versus GAPDH. Gene expressions are represented as mean ±SE comparing post-NMES *versus* pre-NMES. Up-regulated genes have positive values; down-regulated genes have negative values.*

#### Protein Balance

Sarcopenic muscle presents atrophy, which partially depends on reduced anabolic processes, together with increased catabolic processes (Argilés et al., 2015). In this study, various genes related to the protein metabolism were modulated in their expression after NMES training. In particular, the up-regulation of genes involved in the anabolic pathway such as *Insulin like growth factor 1* (*IGF-1*), *Phosphatidylinositol-3-kinase* (*PI3K*), *Mechanistic target of rapamycin* (*MTOR*) and *AKT serine/threonine kinase 1* (*AKT*) in post- *vs* pre-NMES was observed. In parallel, we found that the down-regulated *Forkhead box O1A (rhabdomyosarcoma)* (*FOXO1A*) gene was involved in the catabolic pathway together with *Myostatin (MSTN), Tripartite motif containing 63* (*TRIM63* or *MURF1*), *Proteasome 26S subunit, ATPase 6* (*PSMC6*), *Ubiquitin like modifier activating enzyme 1* (*UBA1*), *Ubiquitin conjugating enzyme E2 A* (*UBE2A*) and *Mitochondrial E3 ubiquitin protein ligase 1* (*MUL1*) genes, involved in the ubiquitin-proteasome degradation system FOXO1A-dependent.

#### Oxidative Management

It is well-recognized that oxidation of biological substrates due to oxidants are generated in the mitochondrial respiratory chain. In particular, such oxidants could have a detrimental effect on elderly muscle myofibers (Fulle et al., 2005; Pietrangelo et al., 2009). Among the endogenous enzymatic systems capable of protecting the cell against oxidative injury, glutathione transferase and glutathione reductase, as well as selenium dependent glutathione peroxidase play a crucial role. Using glutathione (GSH) as a cofactor, glutathione peroxidase reduces H_2_O_2_ to water, converting GSH into its oxidized form (GSSG). Glutathione reductase, in the presence of NADPH, is able to reduce the oxidized glutathione (Mezzetti et al., 1990). NMES caused the down-regulation of the *Glutathione Peroxidase 1* (*GPX1*) gene, encoding a peroxidase protein that detoxifies hydrogen peroxide, thus representing one of the main antioxidant enzymes in humans. A gene which is up-regulated in post-NMES muscle is *Glutathione Reductase* (*GSR*), a fundamental enzyme of the cellular antioxidant defense system that reduces oxidized glutathione disulfide (GSSG) to the reduced form of GSH, being a central cellular antioxidant. Furthermore, we found the *Glutathione S-transferase kappa 1* (*GSTK1*) gene down-regulated. The encoded enzyme catalyzes the conjugation of glutathione to a wide series of hydrophobic substrates aiding the elimination of these compounds from cells.

*Superoxide Dismutase 1, soluble* (*SOD1*) did not vary whereas *Superoxide Dismutase 2, mitochondrial* (*SOD2*), and *Catalase* (*CAT*) genes that encode enzymes that detoxify the cell by $O_{2}^{•-}$ and H_2_O_2_, respectively, were down-regulated by NMES training.

#### Apoptosis

To date, two main apoptotic pathways are known: a death receptor pathway or extrinsic and a mitochondrial pathway or intrinsic (Elmore, 2007). In our study, we found up-regulated genes related to the extrinsic pathway: *Tumor Necrosis Factor* (*TNF), Caspase-8 (CASP8), Caspase-6 (CASP6)*, and *Caspase-7 (CASP7)*, with the exception of *Caspase-3 (CASP3*) and *Fas Associated via Death Domain* (*FADD*) that were slightly down-regulated. On the other hand, it seems that the intrinsic pathway is suppressed, as we found down-regulated C*aspase-9 (CASP9), BCL2 Associated Agonist of cell Death (BAD)*, and *B-cell lymphoma protein 2 (BCL2)* genes. Caspase-9 activation is required for the intrinsic pathway. The regulation and control of these apoptotic events occurs by members of the Bcl-2 protein family that include Bcl-2 and BAD. The *Caspase-2 (CASP2)* gene was also found up-regulated, but its role is not only in apoptosis, but also in cell differentiation (Fava et al., 2012).

#### Myogenesis

After NMES, we found several dysregulated genes involved in the myogenic process. In particular, the *Myogenic Differentiation 1* (*MYOD1*) gene was up-regulated. We also found up-regulated the *Tumor Necrosis Factor* (*TNF*) and the *Mitogen-Activated Protein Kinase 1* (*MAPK1*) an essential signal for myogenic differentiation. *Myogenin* (*MYOG*), is necessary for the fusion of the myogenic precursor cells to either previously existing or new myofibers during the differentiation in the myogenesis process, as well as in the *Paired box 7 (PAX7)*, a transcription factor involved in the regulation of muscle precursor cell proliferation (Boldrin et al., 2010).

### Oxidative Management

#### NBT Assay

**FIGURE 2:**
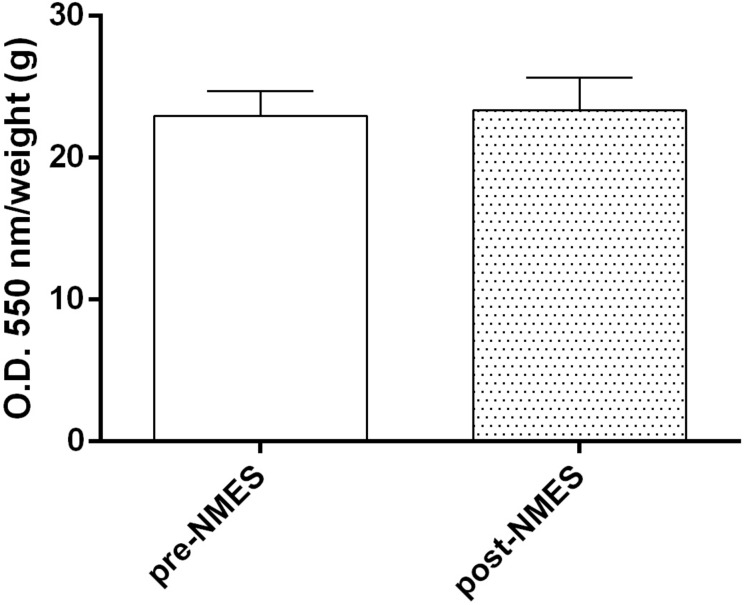
Superoxide anion production. Quantitative analysis of pre-NMES and post-NMES muscle biopsies for $O_{2}^{•-}$. Data (*n* = 5 subjects) are mean ± SEM of three independent experiments in quintuplicates.

Intracellular $O_{2}^{•-}$ levels, revealed by NBT Assay (Figure 2), did not show significant differences (pre-NMES 22.96 ± 1.7; post-NMES 23.36 ± 2.3) between the pre- and post-NMES in muscle samples.

#### Catalase and Superoxide Enzyme Activities

**FIGURE 3:**
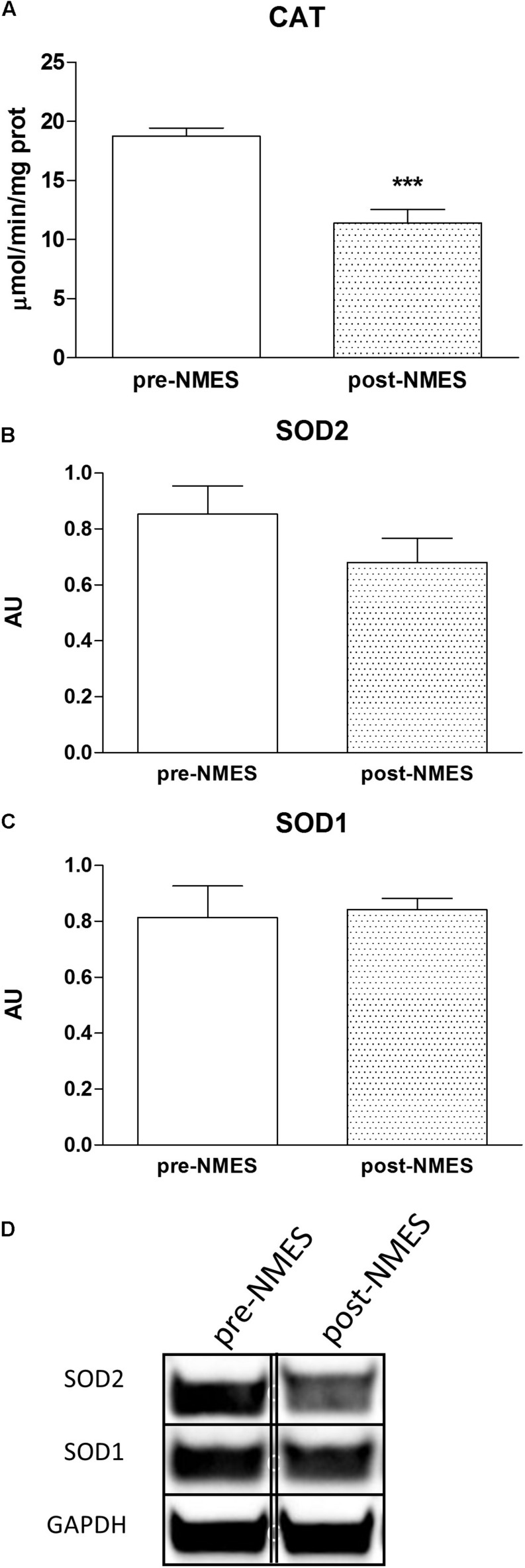
Catalase cytosolic enzymatic activities and SOD Western blotting. In **(A)** is reported the Catalase enzymatic activity in pre- and post-NMES samples as μmol/min/ng prot (*n* = 9). ^∗∗∗^*p* = 0.0005. Data (*n* = 5 subjects) are mean ± SEM of three independent experiments in triplicates. **(B,C)** Show SOD2 and SOD1 densitometric analysis of Western blots performed on four pre-NMES and post-NMES samples expressed as mean ± SD. AU, arbitrary units. The bands (of the same subject) were taken from two nonadjacent lanes originating from exactly the same gel and blot with the same exposure time, but spliced together indicated by double-dotted lines. No change in contrast has been performed. Western Blotting analysis of representative bands of SOD2 and SOD1 **(D)**, of skeletal muscle biopsies pre- and post-NMES.

Figure 3A displays the specific activity of Catalase with a significant decrease in post-NMES in respect to pre-NMES while the SOD1 activity did not vary (data not shown).

#### Protein Expression of SOD1 and SOD2

We analyzed the protein expression of intracellular Super Oxide Dismutases (SOD), distinguishing between the two different forms of SOD, SOD1, and SOD2, cytosolic and mitochondrial protein, respectively. The protein expression was determined on pre- and post-NMES muscle samples using Western Blotting (Figures 3B,C). The SOD2 was slightly, but not statistically nor significantly decreased in POST-NMES samples in respect to the pre-NMES (Figure 3B), while SOD1 did not change (Figure 3C). Representative bands of SOD1 and SOD2 obtained by pre- and post-NMES muscle samples are shown in Figure 3D.

### Gene Expression, Classic Real Time-PCR

**FIGURE 4:**
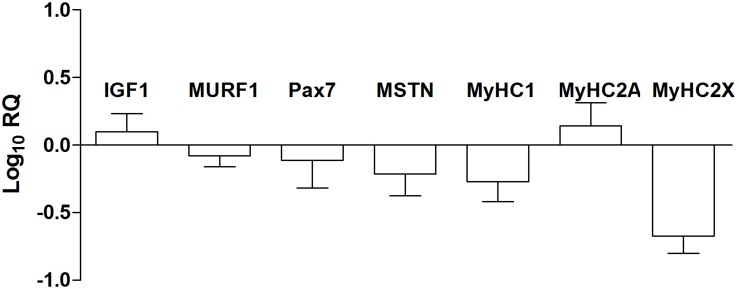
MRFs and MyHC isoforms gene expression. *IGF1, MURF1, PAX7, MSTN, MyHC1, MyHC2A, and MyHC2X* mRNA expression levels in post-NMES muscles *versus* pre-NMES muscles. Data are expressed as the logarithm of Relative Quantification (RQ) of transcripts for these target genes, each versus GAPDH gene expression. Values are expressed as mean ±SEM of three independent experimental sets.

The expression of myogenic transcription factors (*IGF1, Pax7, MURF1*, and *MSTN*) and skeletal muscle myosin heavy chains (*MyHC 1, 2A*, and *2X*) were analyzed by the means of a RT-PCR approach and are reported in Figure 4. Looking at the machinery of myogenic regulatory factors that positively or negatively control the myogenic process, we observed the up-regulation of *Insulin Like Growth Factor 1* (*IGF1)* and *Paired box 7* (*PAX7)* and down-regulation of *Tripartite motif containing 63* (*MURF1)*, and *Myostatin* (*MSTN)*. The fiber type composition of the skeletal muscle is determined by the percentage of slow (MyHC1) and fast (MyHC2A and 2X) myosin heavy chain isoforms. The different isoforms of myosin act as molecular markers that allow the different types of myofibers to be identified. In particular, analyzing the expression of genes (2^–ΔΔCt^) encoding myosin heavy chains, we observed the down-regulation of *MyHC* 1 (pre-NMES 1.0 ± 0.2; post-NMES 0.6 ± 0.3) and *2X* (pre-NMES 1.1 ± 0.5; post-NMES 0.4 ± 0.2) together with an up-regulation of *MyHC2A* (pre-NMES 1.0 ± 0.1; post-NMES 2.5 ± 0.9).

## Discussion

The skeletal muscle of the elderly is characterized by decreased strength, mass and movement velocity, all diagnostic criteria of the status defined as Sarcopenia (Doherty, 2003; Fulle et al., 2004). Physical exercise is considered one of the most effective strategy to slow down muscle aging, especially in terms of mass and function (Negaresh et al., 2019). Besides the classic training, NMES is also a valid method that enhances muscle performance and structure (Acaröz Candan et al., 2019).

Neuromuscular Electrical Stimulation is used to retrieve muscle weakness and to increase muscle strength and hypertrophy in healthy subjects (Wageck et al., 2014; Takano et al., 2016; Langeard et al., 2017; Paillard, 2018). These data are consistent with our study in which a significant increase of isometric strength after a NMES session was shown. Neural adaptations as training-induced changes in the function of the nervous system and afferent feedback to the spinal cord during contractions triggered by NMES would explain, at least in part, the increase of strength of MVC registered at the end of the treatment (Maffiuletti et al., 2006). To investigate the feasibility of the correlation between anthropometric and cellular changes, single myofiber mechanical properties were studied. According to our previous study, (Di Filippo et al., 2017) NMES affected cross-sectional area and force that resulted significantly increased. In particular, this study, shows that the increase in force is due to type II myofibers, and in particular the increase in CSA was due to type IIA myofibers. Thus, the increase in the force of the whole muscle group (MVC) found is based on the increase in the force-developing ability of single myofibers in accordance with previous studies showing similar results (Maffiuletti et al., 2006) therefore likely due to type II myofibers. The modulation of CSA is also in accordance with the down-regulation of MyHC1 and up-regulation of the MyHC2A gene expression.

The skeletal muscle is able to change both structure and function in response to factors that can modify its contractile activity (physical exercise, electrical stimulation, and denervation). These structural and functional adaptations that modify the skeletal muscle phenotype are the result of a rapid variation in the expression of key genes activated or silenced depending on their function.

The myogenic regulatory factor Myogenin is strongly influenced by muscular electrical activity, thus inducing changes in muscle phenotype (Hughes et al., 1999). The increase in MyHC2A and the Myogenin gene expression, with an increase in fast muscle myofiber force that was found, suggests a real metabolic NMES-dependent shift in II type myofibers in the elderly.

The metabolic shift from glycolytic to oxidative that occurs after training is usually accompanied by a modulation of the antioxidant capacity of the cells. The study demonstrates a perturbation of genes that encode the main antioxidant enzymes. Down-regulated glutathione peroxidase and catalase genes that detoxify cells by hydrogen peroxide and transferase that detoxify by metabolites was also found. The catalase enzymatic activity, also results as decreased according to the related gene expression. According to literature on aging (Doria et al., 2012), *SOD1* did not modify its gene and protein expression while *SOD2* tended to decrease both at gene and protein expression levels after NMES training. This result suggests that the SOD2 enzyme that produces mitochondrial superoxide anion, considered, the most dangerous and reactive radical, is less active despite a shift toward an oxidative metabolism. This suggests that NMES induces muscle functional amelioration given to its proper contractions with no increase in muscle oxidative stress. However, further studies could be able to characterize the effect of single bouts of NMES on the redox system inside the muscles of the elderly. This is an important finding, since senescent muscle of the elderly, *per se*, is accompanied by enhanced muscle oxidative stress after physical exercise, and at rest. Another aspect is when macromolecules as proteins are oxidized, they are likely to be degraded by the ubiquitin–proteasome pathway. However, the 24 sessions NMES did not affect the ubiquitin–proteasome pathway as a slight down-regulation of the gene expression of *UBE2A*, *TRIM63*, and *MUL1* was found. Accordingly, we observed an up-regulation of protein synthesis (Stitt et al., 2004) *versus* the protein degradation pathway (Milan et al., 2015) linked to the gene up-regulation of IGF-1, Akt, and mTOR. At the same time, *FOXO1* and *TRIM63* that mediate protein catabolism were found down-regulated.

Skeletal muscle repair, regeneration, growth and remodeling are related to the activation of satellite cells and different local responsive processes with a load-dependent modality (Fulle et al., 2005). The sequential expression of “early” Myogenic Regulatory Factors (MRFs), such as myogenic differentiation factor D (MyoD) and myogenic factor (myf)-5 and “late” MRFs, such as myogenin and myf-6 leads to skeletal muscle repair, regeneration and growth (Kosek et al., 2006). In our study, all MRF genes (*PAX7, MYOD1*, and *MYOG*) were up-regulated, suggesting an activation of the myogenic pathway and of a possible shift toward oxidative phenotype in myofibers of type II (Hughes et al., 1999), while *MSTN*, encodes a protein produced and released by myocytes acting on muscle cells in inhibiting myogenesis, is downregulated. The same results are presented in both classic RT-PCR and in TaqMan low density array (Thomas et al., 2000). The up-regulation of *TNF*, at a physiological level, which activates myogenesis, supports this data (Chen et al., 2007).

Accordingly, with gene expression results improved isometric strength and CSA of type II myofibers were observed, and as a result, an increase in MVC, in accordance to our previous study (Di Filippo et al., 2017).

It could further be argued that the NMES training could induce a hypertrophic effect on skeletal muscle due to the activation of SCs.

Another important pathway found dysregulated by NMES training is apoptosis. In particular, gene expression data revealed that in POST-NMES samples, the extrinsic pathway is activated considering the upregulation of *TNF* as well as genes that encode the initiator (*CASP2*) and executioner (*CASP6* and *CASP7)* at the expense of the intrinsic pathway that seems to be downregulated as *CASP3 and CASP9*. Commonly, apoptosis is associated with muscle degradation, contributing to skeletal muscle atrophy and sarcopenia. Conversely, it was also demonstrated that there is a new role of the apoptotic pathway which is linked to skeletal muscle repair and regeneration (Fulle et al., 2013) as well as to muscle tissue remodeling, following contractile activity (Adhihetty and Hood, 2003). Indeed, In the present study, we can explain that the activation of the apoptotic fiber be can considered in relation to the myogenic process and the progression of differentiation, as demonstrated by previous studies (Fernando et al., 2002). Caspase-8, initiator caspase canonically involved in the “extrinsic pathway”, resulted up-regulated in gene expression analysis in post-NMES, without downstream activation of Caspase3, thus failing the commitment to cell apoptosis. This could indicate the possible role of an apoptotic pathway mediating the differentiation more than cell death (Fulle et al., 2013).

In conclusion, our data demonstrates that NMES is effective on producing physiological adaptation on *Vastus Lateralis* skeletal muscle of active healthy elderly, and in particular:

• increases isometric strength, CSA type II myofibers and, as a result, MVC;
• an activation of both the myogenic pathway and a shift toward oxidative phenotype in myofibers of type II;
• induces muscle functional amelioration with no increase in muscle oxidative stress.
• an apoptotic pathway involved in the differentiation process.

Overall, these results provide new insights for further researches on the elderly who experienced muscle weakening for periodic or permanent immobility.

## Data Availability Statement

Datasets for this study can be found in NCBI using the accession number GSE133720.

## Ethics Statement

The studies involving human participants were reviewed and approved by the Ethic Committee of G. d’Annunzio University. The patients/participants provided their written informed consent to participate in this study.

## Author Contributions

SF and TP conceived and designed the research. RM, ED, MM, CD, VV, LT, LM, and CM performed the experiments. RM, ED, MM, LT, DB, LM, TP, and SF analyzed the data. RM, TP, and SF interpreted the results of experiments. RM, LT, ED, LM, MM, TP, and SF prepared the figures. RM and SF drafted the manuscript. RM, TP, and SF edited and revised the manuscript. RM, LT, ED, MM, CM, LM, DB, CD, VV, TP, and SF approved the final version of the manuscript.

## Conflict of Interest

The authors declare that the research was conducted in the absence of any commercial or financial relationships that could be construed as a potential conflict of interest.

## Footnotes

1 https://www.ncbi.nlm.nih.gov/geo/query/acc.cgi?acc=GSE133720

2 https://www.jamovi.org
